# A time-varying approach to the nexus between environmental related technologies, renewable energy consumption and environmental sustainability in South Africa

**DOI:** 10.1038/s41598-023-32131-4

**Published:** 2023-03-24

**Authors:** Tomiwa Sunday Adebayo, Solomon Eghosa Uhunamure, Karabo Shale

**Affiliations:** 1 Department of Economics, Faculty of Economics and Administrative Science, Cyprus International University, Nicosia, Mersin, Northern Cyprus 10 Turkey; 2 Faculty of Applied Sciences, Cape Peninsula University of Technology, P. O. Box 652, Cape Town, 8000 South Africa

**Keywords:** Climate sciences, Environmental sciences, Energy science and technology

## Abstract

Ecological degradation is a major challenge for all nations. The problem is particularly worrying for South Africa, which has recently suffered from various ecological catastrophes. Thus, the empirical study evaluates the nexus between CO_2_ emissions and financial development, renewable energy, economic growth and environmental-related technologies in South Africa utilizing data between 1980 and 2020. We employed autoregressive distributed lag (ARDL) and time-varying causality to evaluate these connections. The results from the ARDL show that financial development and environmental-related technologies lessen CO_2_ emissions while economic progress intensifies CO_2_ emissions. Surprisingly, renewable energy does not mitigate CO_2_ emissions. Furthermore, the time-varying causality shows that all the independent variables can forecast CO_2_ emissions at different sub-periods. Finally, our results are resilient to various policy ramifications useful in reducing CO_2_ emissions and associated adverse ecological consequences.

## Introduction

Among the biggest difficulties the global economy is currently experiencing are the problems of climate change and ecological deterioration. Threats to human health as well as to productivity and income levels are created by this problem^1,2^. Energy demand increases along with the expansion of economic activities, which increases greenhouse gas emissions that are detrimental to the ecosystem^3^. Practically every country has joined the Paris Agreement and decided to cut GHGs emissions as a way to address the worldwide climate change challenge. The countries’ dedication to accomplishing the aim is crucial to the mission's viability and the accomplishment of the sustainable development goals. Nations are encouraged to pursue green growth in order to cut the trajectory of CO_2_ emissions. As per^4^, the global economy would lose over 18% of its GDP due to climate change.

Regrettably, as stated in the UN Production Gap Report 2021, the present production plan exceeds the cap imposed by the Paris Agreement (United Nations Environment Program, 2021). Thus, all countries must take the necessary steps to guarantee that CO_2_ emissions can be drastically decreased and contribute to achieving sustainable development goals^5–7^. Although many countries have begun to turn to sustainable energy and renewable energy as alternatives to fossil fuels, the quantity needs to be improved to meet the growing energy demand. On the other hand, environmental-related technologies may be a panacea for the environmental issue since it aids in combining sustainable economic growth with improved ecological management^8,9^. This results from positive spillover effects from innovation efforts that lead to the development of new goods, processes, and techniques that can mitigate adverse environmental effects^10,11^. According to^12^, industrialized nations attained higher ecological quality due to cutting-edge technologies that greatly decreased emissions and enhanced ecological conditions.

Even with the expanding body of studies examining the variables that influence environmental quality, the majority of these studies have focused on industrialized nations because most innovation occurs in these nations^13,14^. Nonetheless, given developing nations would be the most impacted by climate change, it is vital for policy measures to comprehend how environmental technologies impact CO_2_ emissions ^15^. The eco-friendly technologies needed to adapt to climate change would differ depending on local situations^12^. Thus, research on emerging nations is crucial for formulating policies supporting environmental technologies.

Considering the efficiency of these variables in achieving carbon neutrality targets, the primary purpose of our research is to concentrate on CO_2_ drivers by evaluating the impact of renewable energy, financial development, and environmental technologies between 1980 and 2020. South Africa is selected for the study due to many reasons. First, developing nations such as South Africa have been overlooked in multiple ecological studies, and few research is accessible. The South African energy system’s cornerstone is coal, which is based on fossil fuels, providing approximately 70% of the installed power production capacity^16^. Nonetheless, the 2019 Integrated Resource Plan outlines a long-term diversification of the power mix by 2030 and strives to reduce the energy sector's carbon impact while supplying more energy and guaranteeing a socioeconomically equitable^16^. The government will have to make difficult decisions as it pursues its goals of diversifying and minimizing the detrimental ecological effect of the nation's energy mix. To do this, it must actively include the public in the discussion. Yet, South Africa's integrated policies, robust regulation, well-designed incentives for low carbon investment, particularly private investment, regional connectivity, and increased efficiency provide it with unmatched capability for the challenge. Based on the interesting facts regarding South Africa, the study formulates the research objectives by asking the following basic questions:

1. Can renewable energy consumption contribute to ecological sustainability in South Africa?
2. Does environmental-related technologies improve ecological sustainability in South Africa?
3. Does financial development improve ecological sustainability in South Africa?

The current study makes three major contributions. First, the present investigation will make a substantial and vital contribution to South Africa's policymakers in light of how climate change and biodiversity affect the country. Second, notwithstanding the expanding literature on technological innovation, the significance of environmental-related innovation has received less attention. Third, the study contributes methodologically by utilizing time-varying causality along with the autoregressive distributed lag model. The implementation of the novel econometric approach such as time-varying causality presents another dimension into these connections. Unlike the conventional causality tests such as^17,18^ causality tests, this technique, which uses a bootstrapping strategy to account for small-sample bias, enables us to identify any structural changes in the framework as well as the progression of causal interrelationships between sub-periods.

The following sections serve as the architecture and design for the present investigation: The assessment of the empirical studies is presented in Sect. 2. Section 3 provides the data and empirical strategies. Section 4 presents the results of the empirical research and their discussion. Section 5 outlines noteworthy policies based on the findings.

## Theoretical framework and literature review

Awareness of the devastating effect of global warming and climate change is no longer new to developing and developed economies. As a result, it is vital to find a long-term solution to this problem. This is evident in the discussions in Paris Agreement, COP26 and COP27, respectively. Nations have come together to find a solution by limiting their level of emissions. Several nations including South Africa have reaffirmed their commitment towards carbon neutrality. However, based on recent development, it looks unlikely that most of these nations will be able to attain their carbon neutrality targets. Several economic variables have been highlighted as the drivers of global warming and climate change. However, proposing a realistic and concrete policy regarding these economic variables' effect on environmental deterioration has faced several roadblocks. Studies have reported dissimilar findings regarding the drivers of ecological decline. Nonetheless, inconclusive results have surfaced which are attributed to the economic conditions of the nation/nations of investigation, techniques employed, and period of study.

With the motive of formulating SDGs policies for Finland^19^, evaluated the driving factors behind ecological deterioration using yearly data between 1990 and 2019. The study applied the novel Fourier approaches in order to propose necessary policies. The study results disclosed that environmental-related technologies and green energy play a vital role towards Finland's carbon neutrality target, while economic growth in Finland is not sustainable. Similarly^14^, used the Fourier approaches in order to evaluate the role of environmental-related technologies and green energy towards the USA carbon neutrality target within the EKC coffin. The investigators used yearly data from 1990 to 2019 to explore the nexus, and the study findings disclosed that ecological-related technologies and green energy lessen CO_2_ emissions while economic progress and financial development deter mitigation of CO_2_ emissions in the United States. Likewise^13^, in their investigation on the drivers of GHG emissions in the Nordic economies, consider the role of energy intensity, eco-innovations, raw material productivity and economic expansion. The investigators assessed the nonlinearity characteristics of the variables and, as a result, employed the panel nonlinear ARDL. The study results disclosed that positive (negative) shifts in energy intensity, eco-innovations and raw material productivity decrease (increase) GHG emissions, while positive (negative) in economic progress increase (decrease) GHG emissions.

The study of^20^ explores the CO_2_ emissions drivers for the 20 biggest economies within the EKC framework. The authors used data from 1990 to 2018 and economic growth, green energy and eco-innovations are considered drivers of CO_2_ emissions. The investigators used MMQR to explore this interrelation and the study findings disclosed that a decrease in CO_2_ emissions is attributed to the intensification of green energy and eco-innovations. In contrast, the intensification of economic expansion and fossil fuel causes an increase in CO_2_. With the aim of devising meticulous policies for the BRICS economies^21^, explore the effect of disintegrated energy, globalization and innovation on ecological footprint using data from 1990 to 2018. The studies used ample techniques-AMG and CCEMG estimators to evaluate this nexus. The study result unearths that mitigation of EF is caused by the intensification of renewable energy, globalization and innovation, while fossil fuel and economic expansion lessen ecological quality.

Likewise, using the Malysia case^22^, used the wavelet and FMOLS and ARDL estimators to analyze the nexus between CO_2_ and financial development, green energy, nonrenewable energy and economic expansion. The findings uncovered that dirty energy and economic expansion intensify CO_2_ emissions while green energy lessens CO_2_ emissions. Besides, an insignificant linkage exists between financial development and emissions, suggesting that Malaysia financial section is still young. Moreover, the study of^23^ for the MINT economies using data between 1990 and 2019 reported that an upsurge in financial development and economic expansion lessens the sustainability of the ecosystem while renewable energy and FDI inflows lessen ecological dilapidation. Furthermore, several studies in energy and environmental literature have reported significant various drivers of ecological quality such as economic growth, financial development, renewable energy, innovation, trade and globalization^24–26^. Likewise, several time series and panel studies also documented substantial studies on the nexus between economic growth, innovation, financial development and CO_2_ emissions^27–33^.

The current investigation explores the determinants of CO_2_ emissions, such as financial development, renewable energy, and environmental related technologies. Drivers of CO_2_ emissions have been investigated in previous research; nevertheless, these studies have certain distinctive gaps or shortcomings. With some appropriate modifications, the present investigation fills these gaps in the previous literature. First, previous researchers have developed a variety of viewpoints on the relationship between CO_2_ emissions, renewable energy, financial development, and environmental technologies. Conversely, the current investigation resolves the literature debate about the correlation between CO_2_ emissions, renewable energy, environmental technologies, and financial progress. The ecological implications of CO_2_ emissions via renewable energy, ecological technologies, and financial development have been studied in a variety of nations, but little is documented about the situation of South Africa. Assessing renewable energy, financial development, environmental technology, and their relationships with CO_2_ emissions in South Africa closes the gap in the literature. Secondly, many techniques and analytical approaches such as ARDL, fully modified OLS (FMOLS), dynamic OLS (DOLS), vector autoregressive (VAR), nonlinear ARDL and dynamic ARDL have been used in the past to investigate the drivers of CO_2_ emissions. Implementing the novel econometric approach, such as time-varying causality, presents another dimension to these connections. Unlike the conventional causality tests such as^17,18^ causality tests, this technique, which uses a bootstrapping strategy to account for small-sample bias, enables us to identify any structural changes in the framework as well as the progression of causal interrelationships between sub-periods. Thus, the current study fills the gap in the ongoing energy and economics literature.

## Data and methods

### Variables source and description

The empirical study evaluates the nexus between CO_2_ emissions and its drivers such as financial development, renewable energy, economic growth and environmental-related technologies in South Africa utilizing data between 1980 to 2020. The dependent is CO_2_, while the determinants of CO_2_ such as financial development, renewable energy, economic growth and related environmental technologies represent the independent variables. The precise and detailed information regarding CO_2_ and the regressors are presented in Table 1.

**Table 1:** Variables and measurement.

Sign	Variable	Measurement	Source
REC	Renewable energy	Renewables per capita (kWh—equivalent)	Ourworldindata database
GDP	Economic growth	GDP Per capita constant US$2015	World Bank database
ETEC	Environmental related technologies	Percentage of all technologies	OECD database
CO_2_	Carbon emissions	Per capita emissions	Ourworldindata database
FD	Financial development	Index	IMF database

The variables of investigation i.e., CO_2_, ETEC, GDP, FD, and REC are logged to ensure conformity to normal distribution in line with the studies of^21,34^. Following the studies of^35,36^, we formulate the following economic function:

1 $${\mathrm{LnCO}}_{2\mathrm{t}}={\beta }_{0}+{\beta }_{1}{GDP}_{t}+{\beta }_{2}{ETEC}_{t}+{\beta }_{3}{REC}_{t}+{\beta }_{4}{FD}_{t}+{\varepsilon }_{t}$$

where; ${\beta }_{0}$ denotes the constant, ${\beta }_{\mathrm{1,2},\mathrm{3,4}, and 5}$ represents the coefficients of the independent variables while ${\varepsilon }_{t}$ represents error term. CO_2_, GDP, ETEC. REC and FD stand for carbon emissions, economic growth, environmental-related technologies, renewable energy and financial development.

**Figure 1:**
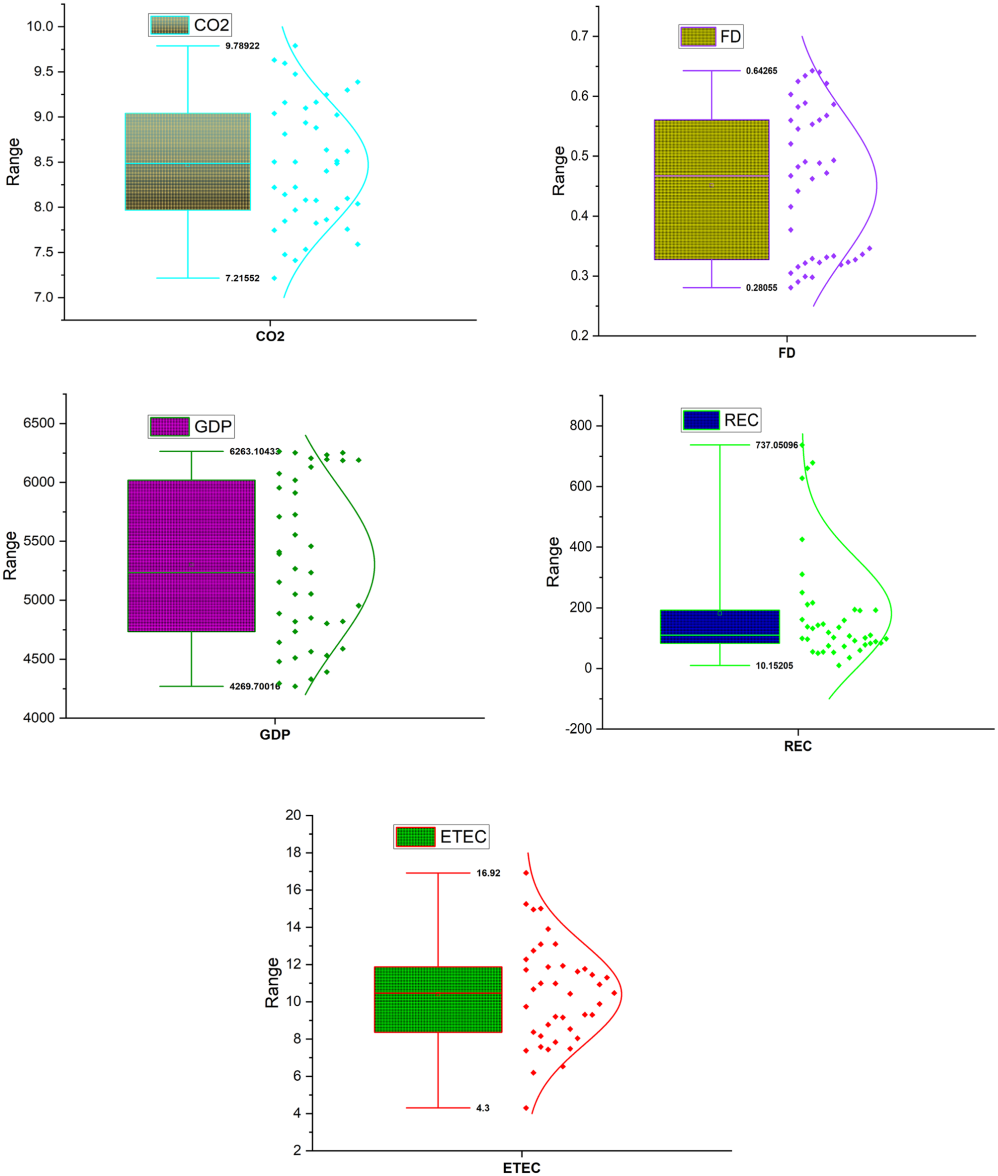
Box Plot.

Table 2 presents the series descriptive statistics. The mean value of GDP (5301.2) is the highest, and it ranges from 4269.7 to 6263.1, REC (181.46) which ranges from 10.152 to 737.05, ETEC (10.404), which ranges between 4.3000 and 16.920, CO_2_ (8.4707) ranges from 7.2155 to 9.7892. The skewness value shows the variables are skewed positively, while the kurtosis value indicates that the variables are platykurtic with REC exemption which is leptokurtic. Figure 1 also presents additional information regarding the variables.

**Table 2:** Descriptive statistics.

	GDP	FD	ETEC	CO_2_	REC
Mean	5301.2	0.4512	10.404	8.4707	181.46
Median	5233.8	0.4672	10.470	8.4854	110.14
Maximum	6263.1	0.6426	16.920	9.7892	737.05
Minimum	4269.7	0.2805	4.3000	7.2155	10.152
Std. Dev	684.39	0.1233	2.7119	0.6879	182.01
Skewness	0.0977	0.0895	0.2150	0.1460	2.0153
Kurtosis	1.5520	1.4839	2.8047	1.9702	5.9708

### Estimation strategies

The ARDL model established by^37^ was employed to evaluate the long-term connectivity between the variables. As a result, the model posits as follows;

2 $$\Delta Ln{CO}_{2t}={\sigma }_{0}+{\sigma }_{1}{\sum }_{i=1}^{p}\Delta L{nCO}_{2t-1}+{\sigma }_{2}{\sum }_{i=1}^{p}\Delta {LnGDP}_{t-1}+{\sigma }_{3}{\sum }_{i=1}^{p}\Delta Ln{ETEC}_{t-1}+{\sigma }_{4}{\sum }_{i=1}^{p}\Delta Ln{REC}_{t-1}+{\sigma }_{5}{\sum }_{i=1}^{p}\Delta Ln{FD}_{t-1}+{\beta }_{1}\Delta Ln{CO}_{2t-1}+{\beta }_{1}\Delta Ln{GDP}_{t-1}+{\beta }_{3}\Delta LnETE{C}_{t-1}+{\beta }_{4}\Delta Ln{REC}_{t-1}+{\beta }_{5}\Delta Ln{FD}_{t-1}+{\varepsilon }_{t}$$

Equation (2) represents the ARDL test's unrestricted ECM. The short-run coefficients shown by ${\sigma }_{1, 2, 3, 4 and 5}$. Moreover, ${\beta }_{1, 2, 3, 4 and 5}$ illustrates the coefficients of the long-run. Also, the long-run cointegration is estimated using the bound test F-statistic value for the Ho hypothesis of "no cointegration," which is denoted by $\mathrm{H}0:{\upbeta }_{1}={\upbeta }_{2}={\upbeta }_{3}={\upbeta }_{4}={\upbeta }_{5}=0$. Furthermore, the null hypothesis is rejected when the bound test F-statistic value surpasses both the higher and lower bound values. Conversely, the Ho hypothesis is deemed valid if the value is smaller. Furthermore, $t-1$ denotes the optimal lag for each variable as calculated by AIC.

The association between the variables is evaluated utilizing the error correction model. To ascertain the short-term characteristics, the lagged terms for each individual coefficient are also used. Furthermore, the error correction term (ECT) enables the collection of information on the long-term dynamics.

3 $$\Delta Ln{CO}_{2t}={\sigma }_{0}+{\sigma }_{1}{\sum }_{i=1}^{p}\Delta L{nCO}_{2t-1}+{\sigma }_{2}{\sum }_{i=1}^{p}\Delta {LnGDP}_{t-1}+{\sigma }_{3}{\sum }_{i=1}^{p}\Delta Ln{ETEC}_{t-1}+{\sigma }_{4}{\sum }_{i=1}^{p}\Delta Ln{REC}_{t-1}+{\sigma }_{5}{\sum }_{i=1}^{p}\Delta Ln{FD}_{t-1}+\varphi EC{T}_{t-1}+{\varepsilon }_{t}$$

The ECT shows short-term alterations while also taking the rate of return to equilibrium into account. Typically, the value of the ECT should range from minus 1 to 0. Furthermore, it ought to be significant and negative. Moreover, the models’ stability is tested utilizing CUSUM and CUSUMSQ.

**Figure 2:**
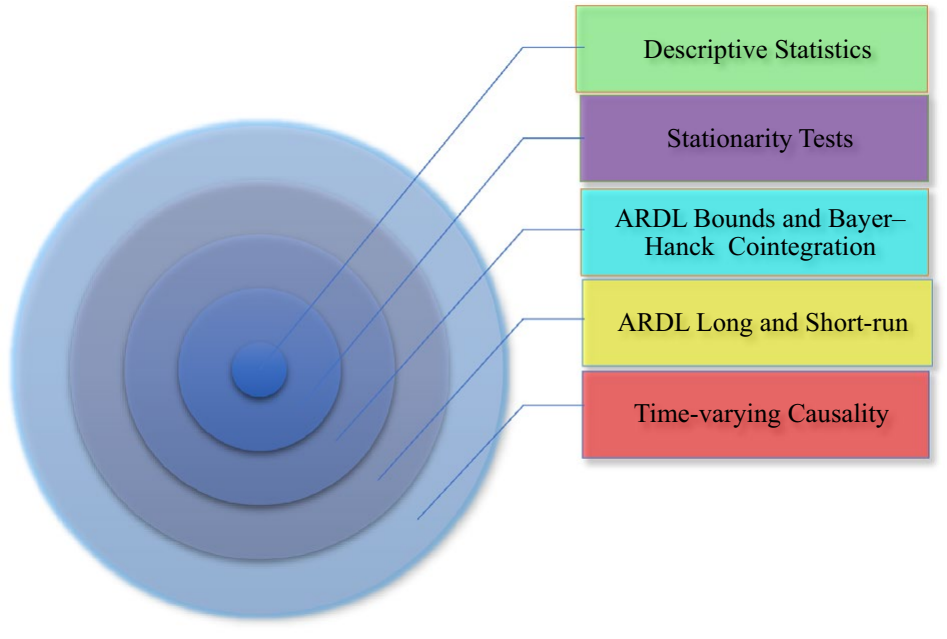
Flow of the study.

Moreover, in line with the studies of^38–40^, we employed the time-varying causality test. Unlike the conventional causality tests such as^17,18^ causality tests, this technique, which uses a bootstrapping strategy to account for small-sample bias, enables us to identify any structural changes in the framework as well as the progression of causal interrelationships between sub-periods. The flow of the study analysis is presented in Fig. 2.

## Findings and discussion

### Preliminary tests

The study evaluates the stationarity features of the series as an initial test (see Table 3). The PP and ADF results documented similar results, showing non-stationarity as level with the exemption of ETEC; Nonetheless, it became stationary after the first difference was taken. In summary, the study series are stationary at mixed order i.e. I(1) and 1(**1).**

**Table 3:** Stationarity test results.

	ADF		PP		ZA			
	I(0)	I(1)	I(0)	I(1)	I(0)	Date	I(1)	Date
LnCO_2_	− 2.283	− 6.420***	− 2.640	− 6.425***	− 4.453	2003	− 5.149***	1993
LnGDP	− 2.388	− 4.740***	− 1.822	− 4.833***	− 3.373	2014	− 5.873***	2015
LnETEC	− 4.545***	−	− 4.475***	–	− 5.106**	1987	–	–
LnREC	− 3.185	− 8.297***	− 3.123	− 8.629***	− 4.638	1992	− 8.794***	1994
LnFD	− 3.197	− 5.289***	− 2.143	− 5.289***	− 4.358	1997	− 5.122***	1996

***P < 1%, **P < 5% and *P < 10%.

### Result of cointegration

Next the study used both ARDL bounds (see Table 4) test and Bayer–Hanck (see Table 5) to capture the long-run dynamics between CO_2_ and its drivers. The results from the bounds test disclosed evidence of long-run dynamics between CO_2_ and its drivers. Moreover, this result is also validated by the Bayer–Hanck Results cointegration. Thus, Ho hypothesis of “cointegration does not exist” is dismissed at 5% as reported by both Bayer–Hanck and ARDL Bounds tests cointegration.

**Table 4:** Bounds test.

	Value	Signif	I(0)	I(1)
F-statistic	3.829**	10%	2.26	3.35
${\mathrm{LnCO}}_{2}=\mathrm{f}(\mathrm{LnGDP},\mathrm{ LnETEC},\mathrm{ LnREC},\mathrm{ LnFD},\mathrm{ Dummy})$	5	5%	2.62	3.79
		1%	3.41	4.68

***P < 1%.

**Table 5:** Bayer–Hanck results.

	Fisher stat	Fisher stat
	EGJOH	EG-JOH-BAN-BOS
${\mathrm{LnCO}}_{2}=\mathrm{f}(\mathrm{LnGDP},\mathrm{ LnETEC},\mathrm{ LnREC},\mathrm{ LnFD},\mathrm{ Dummy})$	20.389***	30.836***
	CV	CV
	10.576	20.143

***P < 1%.

### Results of ARDL

The study explored the drivers of carbon emissions for the case of South Africa using the ARDL, which can identify both short and long run interrelationships (see Table 6). CO_2_ emissions are positively impacted by economic growth in the long-and-short run. Specifically, a 1% upsurge in GDP contributes to the intensification of CO_2_ by 1.128%$\sim$ longrun and 1.1827 $\sim$ short-run. These reinforced the emissions-driven economic growth in South Africa. South Africa is an emerging economy; as a result; they pay close attention to economic progress while little/less attention is assigned to environmental sustainability. It is clear that the growth trajectory of South Africa's economy is not sustainable. Thus, achieving the SDGs will become easier if policy realignment is considered. The studies of^14,20,41^ also reported similar results.

**Table 6:** ARDL results.

Variable	Coefficient	Std. Error	t-Statistic	Prob
Long-run result				
LnGDP	1.1287***	0.2750	4.1030	0.0003
LnETEC	− 0.0772**	0.0268	− 2.8761	0.0105
LnREC	− 0.0069	0.0108	− 0.6399	0.5267
LnFD	− 0.1291***	0.0408	− 3.1590	0.0034
Dummy	0.0021	0.0453	0.0481	0.9619
R^2^	0.93	AdjR^2^	0.92	
Short− run Result				
ECT(-1)	− 0.4942***	0.0694	− 7.1207	0.0000
LnGDP	1.1827***	0.2201	5.3719	0.0001
LnETEC	− 0.0474**	0.0184	− 2.5714	0.0198
LnREC	− 0.0189*	0.0098	− 1.9305	0.0704
LnFD	− 0.2813**	0.1333	− 2.1101	0.0500
Dummy	− 0.1047***	0.0286	− 3.6600	0.0019

***P < 1%, **P < 5% and *P < 10%.

The study also uncovers the negative nexus between environmental-related technologies and CO_2_ emissions in both short and long-run. Specifically, 0.0772%$\sim$ longrun and 0.0474 $\sim$ short run decrease in CO_2_ emissions is caused by a 1% upsurge in South Africa's environmental-related technologies. This shows that related environmental technologies are essential for the decline in CO_2_ emissions, which, as a result, leads to ecological sustainability. As stated by^42^, several nations are investing in environmental related technologies because it is clean and sustainable. Moreover, environmental-related technologies are essential for the energy transition. Thus, South Africa is on the right path toward attaining SDG 7. This result affirmed the studies of^19,43,44^. However, the study of^45^ reported different results by establishing positive interrelationships.

Moreover, renewable energy impacts CO_2_ emissions negatively, though the impact is insignificant. This is a significant issue in the case of South Africa. According to the Paris accord, COP26 and COP27, renewable energy has been highlighted as the solution for achieving sustainable sustainability. Also, renewable energy is secure and long-lasting enough to benefit the environment without slowing down the pace of economic progress. In addition to being sufficient and environmentally benign, renewable energy sources also lessen CO_2_ emissions. This shows that South Africa needs to move towards energy transition swiftly. Moreover, achieving carbon neutrality target is in jeopardy in the case of South Africa. Consequently, policymakers in South Africa must readjust their policies towards achieving SDGs 6 and 7 goals. Our findings align with^46^ studies and^47^ for China, who highlighted an insignificant nexus between CO_2_ and renewable energy. However^35,48,49^ contradict our studies by establishing the declining emissions role of renewable energy.

The study also established the CO_2_ emissions decreasing role of financial development. In specific, the 0.1291%$\sim$ longrun and 0.2813 $\sim$ short run decrease in CO_2_ emissions is caused by a 1% upsurge in financial development in South Africa. The result shows that the financial system of South Africa is mature. The outcome suggests that South Africa’s financial sector embraces sustainable initiatives and prioritizes ecological sustainability. Considerable financial development that addresses ecological concerns helps to reduce CO_2_ emissions. This result complies with the studies of^50,51^. However, this study contradicts^23,52^, who highlighted that with low-interest loans, financial development increases the spending power of the general public. This enables people to buy opulent goods like houses, cars, and air conditioners, all of which put a burden on the environment.

**Figure 3:**
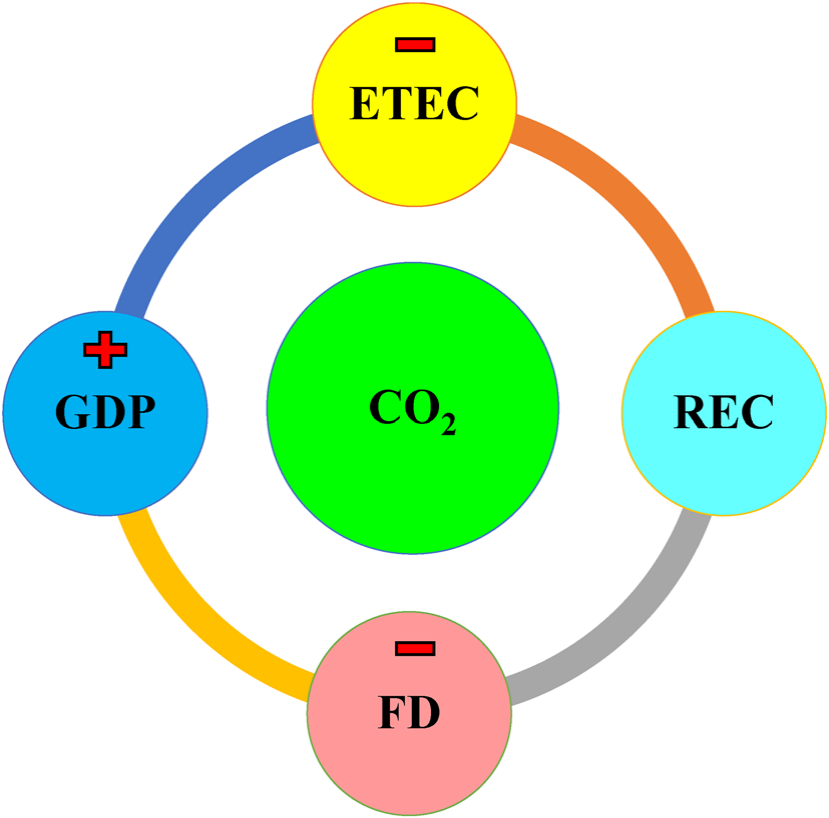
Summary of findings.

Lastly, as expected, the ECT is negative and significant statistically which shows that an earlier adjustment could be rectified in the subsequent periods. Figure 3 shows the summary of the ARDL results.

### Diagnostic test results

**Figure 4:**
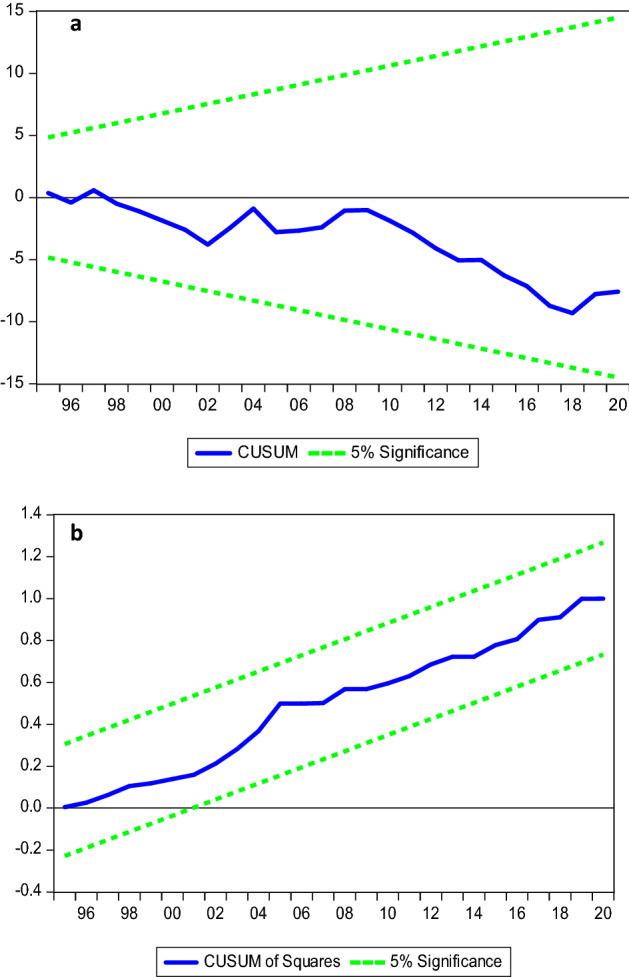
(**a**) CUSUM, (**b**) CUSUM of square.

The study proceeds to the diagnostics tests by evaluating the reliability of the model. Table 7 presents the result of these tests. The results show no evidence of serial correlation issue; residuals are distributed normally, no issue of misspecification, and no problem of heteroskedasticity. Moreover, the CUSUM (see Fig. 4a) and CUSUM of square (see Fig. 4b) support the stability of the model.

**Table 7:** Diagnostics tests.

Test	X^2^	Pvalue
Ramsey RESET test	0.280	0.781
Normality test	0.134	0.934
Heteroskedasticity test:	0.573	0.771
Serial correlation test	0.789	0.459

### Time-varying causality test outcomes

**Figure 5:**
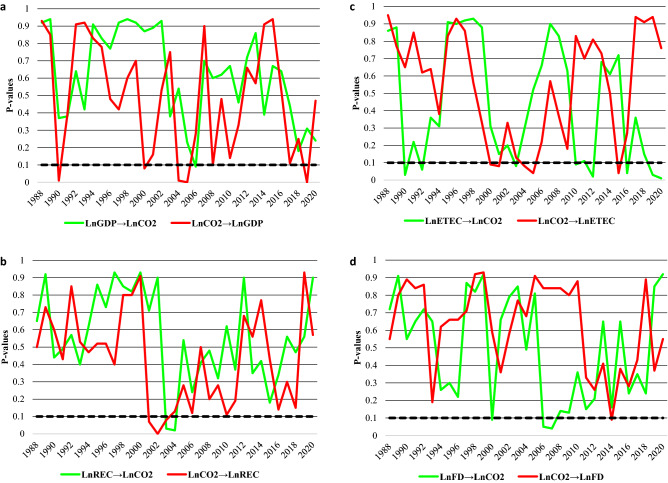
(**a**) Causality between economic growth and CO_2_, (**b**) Causality between renewable energy and CO_2_, (**c**) Causality between related environmental technologies and CO_2_, (**d**) Causality between financial development and CO_2_.

Unlike prior studies that employed the conventional causality tests such as^17,18^ causality tests, we utilized the time-varying causality, which uses a bootstrapping strategy to account for small-sample bias, enables us to identify any structural changes in the framework as well as the progression of causal interrelationships between sub-periods. Figure 5 presents the time-varying causality results between CO_2_ emissions and the regressors. In Fig. 5, the thick black line depicts the 10% level of significance.

Figure 5a presents the causality between CO_2_ and GDP. The results show dismissed Ho hypothesis of "no causality" in 2007. On the flip side, the null hypothesis of "no causality" from CO_2_ emissions to GDP was dismissed in 1990, 2000, 2004–2006, 2008 and 2019. In summary, feedback causality exists between GDP and CO_2_ as shown by the time-varying causality. Figure 5b unveil causality between CO_2_ and renewable energy. Evidence of causality from renewable to CO_2_ is noticed from 2003–2004 while causality from CO_2_ to renewable energy is captured from 2001 to 2003. Figure 5c shows causality between environmental-related technologies and CO_2_. Specifically, causality surfaced from environmental technologies to CO_2_ from 1990–1992, 2003, 2011–2012, 2017 and 2019. Conversely, causality emerged from CO_2_ to environmental technologies from 2000–2001, 2004–2006 and 2015. Lastly, Fig. 5d display the causality between financial development and CO_2._ The Ho hypothesis of “no causality” is refuted in 2000 and 2006–2008 from financial development to CO_2_. On the flipside, Ho hypothesis of “no causality” from CO_2_ to financial development was refuted in 2014.

## Conclusion and policy remarks

The empirical research pursues to evaluate the nexus between CO_2_ emissions and its drivers-financial development, renewable energy, economic growth and environmental-related technologies in South Africa utilizing data between 1980 to 2020. This investigation seeks to uncover this association more extensively, meticulously, and analytically to contribute to South Africa's CO_2_ reduction measures. In doing so, we employed autoregressive distributed lag (ARDL) and time-varying causality to evaluate these connections. The results from the ARDL show that financial development and environmental technologies lessen CO_2_ emissions while economic progress intensifies CO_2_ emissions. Surprisingly, renewable energy does not mitigate CO_2_ emissions. Furthermore, the time-varying causality shows that all the dependent variables can forecast CO_2_ emissions at sub-periods.

The findings of our study have a variety of significant policy recommendations. Environmental-related technologies help to reduce carbon emissions. Hence, the South African government should emphasize ecological innovations and acknowledge the significance of environmental-related technologies in lowering CO_2_ emissions. To address a worsening of the ecosystem brought on by CO_2_ emissions, governments and policymakers in South Africa must develop measures to increase investment in eco-friendly technologies. Therefore, the South African government should launch new research and development initiatives in environmentally friendly technologies, and authorities should collaborate with industry to create cutting-edge initiatives to combat the largest damaging factor—CO_2_ emissions from business operations. Furthermore, investors should be trained to invest in companies that are making significant efforts to reduce their adverse environmental impact by implementing eco-friendly technologies. Moreover, strategies should be developed and implemented to promote the corporate sector to take such actions.

South Africa relies far too heavily on fossil fuels. Resource and pollution taxes are two examples of carbon-emitting resource levies incentivizing consumers and companies to use renewable energy sources. Investors and customers must favor eco-friendly goods and services. Moreover, energy sources that emit CO_2_ should be substituted with renewables. Additionally, since diesel cars generate a lot of CO_2_ and fine particulates, high vehicle registration and circulation costs can prevent people from buying them. Furthermore, South Africa should shift to electromobility in the automobile industry and other transportation methods. These measures will not only help South Africa create a sustainable ecosystem but will also aid in transforming the nation's economy in favor of the environment.

The present study, like many others, has constraints. Due to data restrictions, we could not incorporate several other relevant drivers in our research, such as globalization, economic complexity, government stability, political risk, human capital, etc. Thus, future studies should incorporate those variables to assist the nation’s policymakers.

## Data Availability

Data are readily available from https://data.worldbank.org/country/south-africa and https://ourworldindata.org/.

## References

[1] Ozturk I, Acaravci A (2016). Energy consumption, CO2 emissions, economic growth, and foreign trade relationship in Cyprus and Malta. Energy Sources Part B.

[2] Zhang Q, Shah SAR, Yang L (2022). Modeling the effect of disaggregated renewable energies on ecological footprint in E5 economies: Do economic growth and R&D matter?. Appl. Energy.

[3] Adebayo TS (2023). Trade-off between environmental sustainability and economic growth through coal consumption and natural resources exploitation in China: New policy insights from wavelet local multiple correlation. Geol. J..

[4] Swiss Re Institute (2023). Swiss Re Institute Estimates USD 83 Billion Global Insured Catastrophe Losses in 2020, The Fifth-Costliest On record.

[5] Naqvi SAA, Shah SAR, Anwar S, Raza H (2021). Renewable energy, economic development, and ecological footprint nexus: Fresh evidence of renewable energy environment Kuznets curve (RKC) from income groups. Environ. Sci. Pollut. Res..

[6] Shah SAR, Naqvi SAA, Riaz S, Anwar S, Abbas N (2020). Nexus of biomass energy, key determinants of economic development and environment: A fresh evidence from Asia. Renew. Sustain. Energy Rev..

[7] Shah SAR, Naqvi SAA, Nasreen S, Abbas N (2021). Associating drivers of economic development with environmental degradation: Fresh evidence from Western Asia and North African region. Ecol. Indic..

[8] Costantini V, Crespi F, Marin G, Paglialunga E (2017). Eco-innovation, sustainable supply chains and environmental performance in European industries. J. Clean. Prod..

[9] Ding Q, Khattak SI, Ahmad M (2021). Towards sustainable production and consumption: Assessing the impact of energy productivity and eco-innovation on consumption-based carbon dioxide emissions (CCO2) in G-7 nations. Sustain. Prod. Consum..

[10] Ahmad M (2021). Modelling the dynamic linkages between eco-innovation, urbanization, economic growth and ecological footprints for G7 countries: Does financial globalization matter?. Sustain. Cities Soc..

[11] Huang Y, Ahmad M, Ali S, Kirikkaleli D (2022). Does eco-innovation promote cleaner energy? Analyzing the role of energy price and human capital. Energy.

[12] Popp D (2012). The role of technological change in green growth. Environ. Res. Econ. Environ..

[13] Alola AA, Adebayo TS (2022). Are green resource productivity and environmental technologies the face of environmental sustainability in the Nordic region?. Sustain. Dev..

[14] Kirikkaleli D, Sofuoğlu E, Ojekemi O (2023). Does patents on environmental technologies matter for the ecological footprint in the USA? Evidence from the novel Fourier ARDL approach. Geosci. Front..

[15] Acheampong AO (2018). Economic growth, CO2 emissions and energy consumption: What causes what and where?. Energy Econ..

[16] IEA (2023). International Energy Association South Africa.

[17] Toda HY, Yamamoto T (1995). Statistical inference in vector autoregressions with possibly integrated processes. J. Econom..

[18] Granger CWJ (1969). Investigating causal relations by econometric models and cross-spectral methods. Econometrica.

[19] Alola AA, Adebayo TS (2023). The potency of resource efficiency and environmental technologies in carbon neutrality target for Finland. J. Clean. Prod..

[20] Çitil M (2023). Does green finance and institutional quality play an important role in air quality. Environ. Sci. Pollut. Res..

[21] Ojekemi OS, Rjoub H, Awosusi AA, Agyekum EB (2022). Toward a sustainable environment and economic growth in BRICS economies: Do innovation and globalization matter?. Environ. Sci. Pollut. Res..

[22] Zhang L (2021). Modeling CO2 emissions in Malaysia: An application of Maki cointegration and wavelet coherence tests. Environ. Sci. Pollut. Res..

[23] Ağa M, Agyekum EB, Kamel S, El-Naggar MF (2022). Do renewable energy consumption and financial development contribute to environmental quality in MINT nations? Implications for sustainable development. Front. Environ. Sci..

[24] Shah SAR, Naqvi SAA, Anwar S, Shah AA, Nadeem AM (2022). Socio-economic impact assessment of environmental degradation in Pakistan: Fresh evidence from the Markov switching equilibrium correction model. Environ. Dev. Sustain..

[25] Zhang Q, Naqvi SAA, Shah SAR (2021). The contribution of outward foreign direct investment, human well-being, and technology toward a sustainable environment. Sustainability.

[26] Zhang Q, Shah SAR, Yang L (2022). An appreciated response of disaggregated energies consumption towards the sustainable growth: A debate on G-10 economies. Energy.

[27] Agyekum EB (2022). Another look at the nexus between economic growth trajectory and emission within the context of developing country: Fresh insights from a nonparametric causality-in-quantiles test. Environ. Dev. Sustain..

[28] Bamidele R, Ozturk I, Gyamfi BA, Bekun FV (2022). Tourism-induced pollution emission amidst energy mix: Evidence from Nigeria. Environ. Sci. Pollut. Res..

[29] Bamidele RO, Ozturen A, Haktanir M, Ogunmokun OA (2023). Realizing Green Airport Performance through Green Management Intransigence, Airport Reputation, Biospheric Value, and Eco-Design. Sustainability.

[30] Bekun FV, Gyamfi BA, Bamidele RO, Udemba EN (2022). Tourism-induced emission in Sub-Saharan Africa: A panel study for oil-producing and non-oil-producing countries. Environ. Sci. Pollut. Res..

[31] Eweade BS, Uzuner G, Akadiri AC, Lasisi TT (2022). Japan energy mix and economic growth nexus: Focus on natural gas consumption. Energy Environ..

[32] Ramzan M, Abbasi KR, Iqbal HA, Adebayo TS (2023). What’s at Stake? The empirical importance of government revenue and debt and renewable energy for environmental neutrality in the US economy. Renew. Energy.

[33] Yang C, Song X (2023). Assessing the determinants of renewable energy and energy efficiency on technological innovation: Role of human capital development and investement. Environ. Sci. Pollut. Res..

[34] Shahbaz M, Nasir MA, Roubaud D (2018). Environmental degradation in France: The effects of FDI, financial development, and energy innovations. Energy Econ..

[35] Adedoyin FF, Ozturk I, Agboola MO, Agboola PO, Bekun FV (2021). The implications of renewable and non-renewable energy generating in Sub-Saharan Africa: The role of economic policy uncertainties. Energy Policy.

[36] Adewale Alola A, Ozturk I, Bekun FV (2021). Is clean energy prosperity and technological innovation rapidly mitigating sustainable energy-development deficit in selected sub-Saharan Africa? A myth or reality. Energy Policy.

[37] Pesaran MH, Shin Y, Smith RJ (2001). Bounds testing approaches to the analysis of level relationships. J. Appl. Econom..

[38] Balcilar M, Bekiros S, Gupta R (2017). The role of news-based uncertainty indices in predicting oil markets: A hybrid nonparametric quantile causality method. Empir. Econ..

[39] Ramzan M, Razi U, Quddoos MU, Adebayo TS (2022). Do green innovation and financial globalization contribute to the ecological sustainability and energy transition in the United Kingdom? Policy insights from a bootstrap rolling window approach. Sustain. Dev..

[40] Irfan M, Sunday Adebayo T, Cai J, Dördüncü H, Shahzad F (2022). Analyzing the mechanism between nuclear energy consumption and carbon emissions: Fresh insights from novel bootstrap rolling-window approach. Energy Environ..

[41] Awosusi AA (2022). The sustainable environment in Uruguay: The roles of financial development, natural resources, and trade globalization. Front. Environ. Sci..

[42] Oyebanji MO, Kirikkaleli D, Ayobamiji AA (2023). Consumption-based CO2 emissions in Denmark: The role of financial stability and energy productivity. Integr. Environ. Assess. Manag..

[43] Sun Y, Yesilada F, Andlib Z, Ajaz T (2021). The role of eco-innovation and globalization towards carbon neutrality in the USA. J. Environ. Manage..

[44] Wang L, Chang H-L, Rizvi SKA, Sari A (2020). Are eco-innovation and export diversification mutually exclusive to control carbon emissions in G-7 countries?. J. Environ. Manage..

[45] Adebayo TS, Kirikkaleli D (2021). Impact of renewable energy consumption, globalization, and technological innovation on environmental degradation in Japan: Application of wavelet tools. Environ. Dev. Sustain..

[46] Alola AA, Adebayo TS, Onifade ST (2022). Examining the dynamics of ecological footprint in China with spectral Granger causality and quantile-on-quantile approaches. Int. J. Sustain. Dev. World Ecol..

[47] Pata UK (2018). Renewable energy consumption, urbanization, financial development, income and CO2 emissions in Turkey: Testing EKC hypothesis with structural breaks. J. Clean. Prod..

[48] Samour A, Baskaya MM, Tursoy T (2022). The impact of financial development and FDI on renewable energy in the UAE: A path towards sustainable development. Sustainability.

[49] Acheampong AO, Adams S, Boateng E (2019). Do globalization and renewable energy contribute to carbon emissions mitigation in Sub-Saharan Africa?. Sci. Total Environ..

[50] Rahman MM, Alam K (2022). Impact of industrialization and non-renewable energy on environmental pollution in Australia: Do renewable energy and financial development play a mitigating role?. Renew. Energy.

[51] Ling G, Razzaq A, Guo Y, Fatima T, Shahzad F (2022). Asymmetric and time-varying linkages between carbon emissions, globalization, natural resources and financial development in China. Environ. Dev. Sustain..

[52] Afshan S, Yaqoob T (2022). The potency of eco-innovation, natural resource and financial development on ecological footprint: A quantile-ARDL-based evidence from China. Environ. Sci. Pollut. Res..

